# Surveillance of the short-term impact of fine particle air pollution on cardiovascular disease hospitalizations in New York State

**DOI:** 10.1186/1476-069X-8-42

**Published:** 2009-09-22

**Authors:** Valerie B Haley, Thomas O Talbot, Henry D Felton

**Affiliations:** 1 Bureau of Environmental and Occupational Epidemiology, New York State Department of Health, Albany, NY, USA; 2 Bureau of Air Quality Surveillance, New York State Department of Environmental Conservation, Albany, NY, USA

## Abstract

**Background:**

Studies have shown that the effects of particulate matter on health vary based on factors including the vulnerability of the population, health care practices, exposure factors, and the pollutant mix.

**Methods:**

We used time-stratified case-crossover to estimate differences in the short-term impacts of PM_2.5 _on cardiovascular disease hospital admissions in New York State by geographic area, year, age, gender, co-morbid conditions, and area poverty rates.

**Results:**

PM_2.5 _had a stronger impact on heart failure than other cardiovascular diagnoses, with 3.1% of heart failure admissions attributable to short-term PM_2.5 _exposure over background levels of 5 ug/m^3^. Older adults were significantly more susceptible to heart failure after short-term ambient PM_2.5 _exposure than younger adults.

**Conclusion:**

The short-term impact of PM_2.5 _on cardiovascular disease admissions, and modifications of that impact, are small and difficult to measure with precision. Multi-state collaborations will be necessary to attain more precision to describe spatiotemporal differences in health impacts.

## Introduction

Numerous studies have shown an adverse relationship between exposure to ambient fine particulate matter (PM_2.5_) and cardiovascular health [1,2]. The short-term impact, which can be measured using readily available health outcome and air pollution data, has been shown to vary by region, with cardiovascular risks higher in counties located in the Eastern U.S. [3]. These geographic differences may be related to differences in the demographic composition and health status of the populations, exposure factors, and the pollutant mix [4-7]. Clarification of the individual and group-level factors associated with increased susceptibility to air pollution is important to guide further research into the biologic mechanisms of action, support risk assessments that inform National Ambient Air Quality Standards (NAAQS), and develop public health interventions.

The Environmental Public Health Tracking Program (EPHT, http://www.cdc.gov/nceh/tracking/) is collecting and integrating nationally consistent health and environmental data to describe patterns and relationships of environmental hazards and disease, identify populations at risk, implement and evaluate interventions, and reduce environmentally related diseases. In some areas of New York State (NYS), ambient particulate matter concentrations are high; in the metropolitan area of New York City (NYC) ten counties are out of compliance with the Annual PM_2.5 _NAAQS [8]. NYS also has the highest proportion of deaths (34.4%) caused by diseases of the heart, compared to the other states [9]. For these reasons, the NYS EPHT program wanted to obtain estimates of the extent to which air pollution may be affecting cardiovascular health, and identify particularly sensitive subpopulations using local data.

Studies of the modifying effects of socioeconomic status (SES) have been mixed, and dependent on the geographic resolution of the SES variables [10]. Most studies of short-term health impacts have examined SES at the city or county level. The NYS hospital data have greater geographic accuracy than typically employed in air pollution epidemiology because they identify individuals at the street address rather than the county level. This allows examination of the impact of poverty at the census tract level. To estimate ambient PM_2.5 _concentrations at a higher spatial and temporal resolution than could be achieved with Federal Reference Method Monitors (FRMs), we incorporated data from Tapered Element Oscillating Microbalance (TEOM) method samplers [11]. Few health studies have used data from these newer, automated hourly PM_2.5 _samplers [12,13].

## Methods

### Health data

We obtained a database of all persons discharged from New York State hospitals between 2001 and 2005 with a primary diagnosis of cardiovascular disease from the New York Statewide Planning and Research Cooperative System. This system contains billing and medical abstract information from all hospitals except Federal and Veterans Administration hospitals. NYSDOH Institutional Review Board and Data Protection Review Board approvals were obtained to access individually identifying information such as address, date of birth, and date of admission.

We geocoded the data using MapMarker version 13 (PB MapInfo Corp, Troy, NY). Approximately 90% of hospital admissions were geocoded to the street address, 1.5% to ZIP+2 or ZIP+4 centroids, and 8.5% were assigned to population-weighted ZIP code centroids; less than 0.05% had invalid ZIP codes and were excluded. Several other exclusions were applied as well. We deleted 0.7% of cases that had AIDS because addresses and admission dates were not available, 33.8% of admissions that originated from outside the emergency room (e.g. transfers, scheduled admissions) because the timing of these admissions would be less related to the current day's air pollution, 2.3% of cases under age 35 due to potentially different disease characterization, and 6.2% of cases that were readmitted less than 28 days since the last admission for statistical independence within the case-crossover design.

Information on poverty was obtained from the 2000 U.S. Census by ZIP code tabulation area (ZCTA) and census tract. We calculated the percentage of adults living below the poverty level, which has been identified as a simple but robust indicator of socioeconomic gradient [14]. Census tract data were used for the 90% of cases geocoded better than ZIP code centroids; the remaining 10% were assigned ZCTA poverty level. This area level poverty rate was assigned to each case through a geographic link with the point case location.

### Air data

PM_2.5 _is measured for regulatory purposes using the FRM. In this method, a sample is collected on a filter for 24 hours at ambient temperature and humidity. Eight to 177 hours later, the filter is transported to a lab to be weighed. The 24-hour mass concentration of PM_2.5 _is calculated as the difference in filter weight before and after sampling, divided by the volume of sampled air. Due to time and costs entailed in collecting and analyzing the samples, measurements are usually taken only every three days at most monitored stations. However, one day of elevated air pollution concentrations may be related to health effects distributed over several days due to the latency period between the exposure, the resulting biological event, and time to observation, so daily data are needed to efficiently measure this distributed lag effect.

Newer automated TEOMs (TEOM 1400AB, ThermoFisher, Franklin, MA) provide PM_2.5 _concentrations every hour. TEOMs operate by collecting particulate matter on a small filter on the end of an oscillating tube. The concentration of PM_2.5 _is determined based on the relationship between the change in frequency of oscillation and the mass of material collected. TEOMs can be operated in more locations and at a lower cost than FRMs, however, they may not currently be used in determining compliance with NAAQS.

In areas with significant seasonal temperature changes like New York State, there is a seasonal bias in the difference between TEOM and FRM measurements. In general, TEOM monitors that utilize a mass sensor heated to 50 deg C measure less PM_2.5 _in colder months due to the loss of semi-volatile compounds. FRMs also lose a portion of the collected semi-volatile compounds through evaporation and chemical reactions as the filters sit in the samplers awaiting collection and are transported to the lab. PM_2.5 _in the Northeast is primarily nonvolatile and highly correlated using the two methods, after adjusting for seasonal differences. Thus, data from the two methods can be combined, after adjusting for the seasonal variation, to obtain daily estimates of PM_2.5 _at a larger number of locations than could be obtained with FRMs alone, and to use exposure estimates that are FRM-like, and therefore consistent with the majority of health studies in the literature.

**Figure 1:**
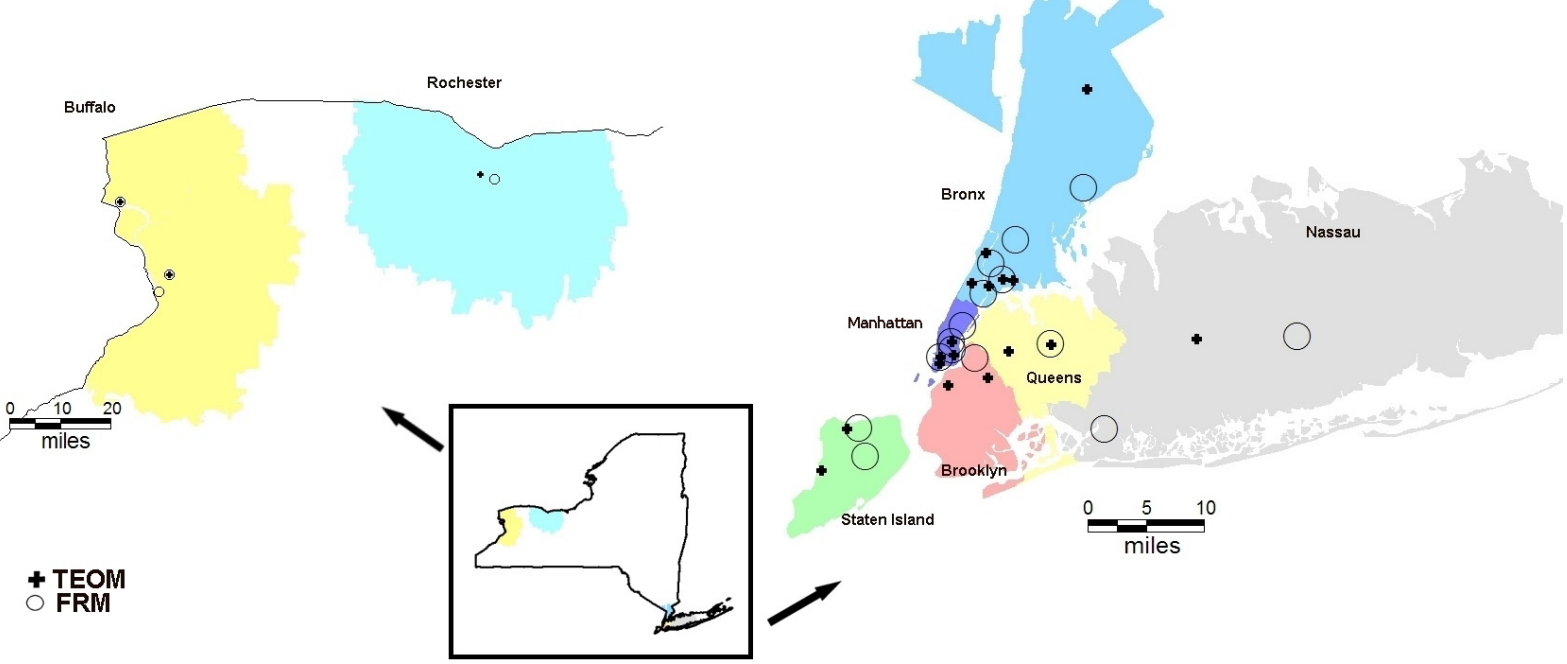
**Map of Study Area**. TEOM = Tapered Element Oscillating Microbalance Monitor. FRM = Federal Reference Method Monitor

We obtained hourly TEOM and 24-hour average FRM PM_2.5 _measurements for the period 2001 through 2005 from the NYS Department of Environmental Conservation and EPA. We summarized the hourly TEOM data into 24-hour averages if at least 75% of the data were available for the day. We selected all TEOM and FRM monitors that were approximately 75% complete for the study period of interest. This resulted in a selection of 20 TEOM monitors and 19 collocated or nearby FRM monitors. The locations of the monitors are shown in Figure 1.

Using two FRM monitors with daily measurements, we developed a model for the FRM/TEOM relationship based on every third day of data to apply to all 20 monitors. For each day with a TEOM measurement, the adjusted TEOM measurement was calculated to be the original TEOM measurement times the 21 day moving average of the ratio of FRM/TEOM. The 21 day centered moving average accounts for the day-of-week effect of air pollution because each day of the week is sampled the same number of times. On average, the TEOMs and FRMs were correlated with an r-squared of 0.86 before the adjustment procedure and 0.92 after the adjustment procedure.

Correlations between the monitors were evaluated to aid in dividing the monitors into geographically similar areas. In the Buffalo, Rochester, and Nassau areas, 25 mile buffers were created around the groups of monitors. In the NYC metropolitan area smaller buffers were used since there is a dense network of monitors in this area. The eight study areas, (Buffalo, Rochester, Bronx, Manhattan, Staten Island, Brooklyn, Queens, and Nassau: shown in different colors in Figure 1), cover approximately 70% of the population of New York State. Cases were selected for analysis if their ZIP code centroids fell within these 8 air pollution regions. All of the monitors within each area were mean adjusted to preserve the day-to-day variation of the monitors, such that missing values on any one day would not bias the daily average. In each air pollution region, daily weighted averages were calculated by weighting FRMs three times more strongly than TEOMs, since FRM data are more consistent with other studies. After this procedure, there remained 2.7% missing data. These remaining missing values were imputed using mean-adjusted data from one of the other nearby air pollution regions.

Temperature, humidity, and air pressure data were obtained from the National Climatic Data Center (NCDC). The heat index was calculated based on temperature and humidity. Data on large snow storms were obtained from the NCDC Storm Events Database. The closest weather monitor data was assigned to each region.

### Method of analysis

We used time-stratified case-crossover analysis [15-17] to assess the effect of PM_2.5 _on the risk of hospitalizations. This method compares the air quality just before someone enters the hospital, with the air quality at reference times within the same prespecified stratum of time, when the person is not hospitalized. Since each case serves as its own control, many important slowly varying personal characteristics that could be confounders, such as socioeconomic factors and smoking, are controlled for by design. We used 28-day strata, and compared cases with referent times 7, 14, and 21 days before or after the case, within the same stratum. We made the comparison on the same day of the week to control for personal activity patterns. This design sampled three referent days for every case. There were 64 strata, from January 20, 2001 to December 16, 2005. To meet the assumption of independent events within each stratum, we implemented a 28-day washout period on the entire dataset of cardiovascular discharges. We estimated the relative increase or decrease in the risk of hospitalization per unit change in pollutant concentration using conditional logistic regression, in SAS statistical software version 9.1 (SAS Institute, Cary, NC). The attributable risk of the short-term exposure was estimated directly using the distribution of exposure in the cases [18].

We developed a statewide case-crossover model for the effect of PM_2.5 _on each of five categories of cardiovascular disease: ischemic heart disease (ICD-9 codes 410-414), heart rhythm and conduction disturbances (426, 427), heart failure (428), cerebrovascular disease (430-438), and peripheral artery disease (440-448). The categories were analyzed separately to investigate which diagnoses were most strongly related to PM_2.5_, and because the admission rates had different seasonal trends, indicating different mechanisms. The models were all adjusted with average apparent temperature as linear and quadratic terms on lags 0 and 1, average barometric pressure on lags 0 and 1, holidays, the day after holidays, and major snow storms. There was a small amount of correlation (r = 0.55) between adjacent days, causing some instability in the PM_2.5 _estimates in an unconstrained distributed lag model. Therefore, a constrained quadratic polynomial distributed lag model [19] was modeled across lags 0 to 4 to examine the temporal impact of PM_2.5 _on admissions.

Measurement of significant modification of the main effect is most likely when the main effect is itself significant. Thus, a detailed analysis of effect modification is only presented for heart failure cases, which had the strongest association with PM_2.5_. Since the effect of PM_2.5 _was fairly consistent over the course of lags 0 to 2, further models used the average PM_2.5 _concentration over days 0 to 2, reducing the number of possible parameters in the model. Heterogeneity in the effect of PM_2.5 _among the eight study areas was assessed by combining the eight stratified estimates using inverse variance weighting, and calculating the I^2 ^statistic [20]. Effect modification was modeled using a variable representing the main effect (i.e. PM_2.5_), and a variable representing the interaction (i.e. PM_2.5 _multiplied by an indicator variable for the condition of interest). The results were converted to the risk scale by exponentiating the parameters of interest, i.e. exp(main effect slope plus interaction slope). At the individual level we tested patient age (in approximate quartiles), gender, and co-morbid conditions such as diabetes and atherosclerosis from individual-level data on the hospitalization files. Identifiers on the hospital records allowed us to include co-morbid conditions only if they were present before admission. At the group level, we tested for modification by census tract poverty rates (in tertiles), season, and year. Interaction was assessed by examining the significance of the interaction terms.

We conducted a sensitivity analyses to determine whether the basic model results were robust to the selection of controls, comparing the aforementioned design that sampled referent times +/- 7, 14, and 21 days within 28-day strata, to a design with referent times +/- 7 and 14 days within 21-day strata.

## Results

**Figure 2:**
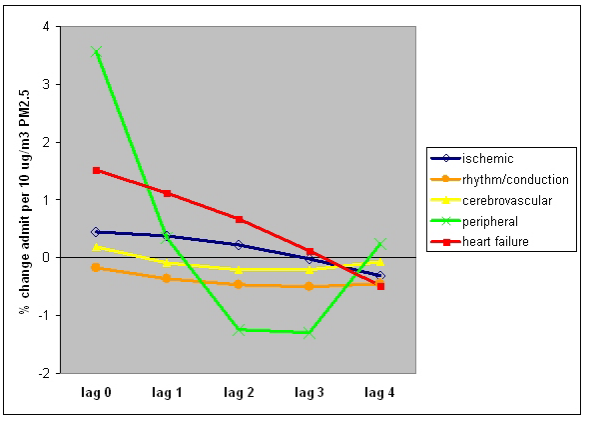
**Distributed lag effect of PM_2.5 _over 5 days, by diagnosis group**.

During the study period of January 20, 2001 to December 16, 2005, there were 647,830 cardiovascular disease admissions from the emergency room, 33% with a primary diagnosis of ischemic heart disease, 26% heart failure, 21% cerebrovascular, 17% heart rhythm/conduction, and 2% peripheral artery disease. The mean distributed lag effect is plotted by disease category in Figure 2, and summarized in Table 1. The largest cumulative effect was observed for heart failure admissions; the risk was significantly increased for lags 0 to 2. The strongest immediate effect of air pollution was found on peripheral disease with a significantly increased risk on lag 0, but cumulatively the risk was not as great as for heart failure. The negative association of peripheral disease admissions with PM_2.5 _levels 2 and 3 days before the event could be due to the instability of the prediction given the relatively small number of cases, or a harvesting effect, meaning that these highly susceptible individuals were removed from the pool of potential cases.

**Table 1:** Percent increased risk (and 95% confidence interval) of hospital admission per 10 ug/m^3 ^PM_2.5 _from distributed lag model, by diagnosis group

**Lag Day**	**Ischemic HD**	**Rhythm/Conduction**	**Cerebrovascular**	**Peripheral**	**Heart failure**
0	0.456 (-0.323, 1.240)	-0.174 (-1.253, 0.916)	0.186 (-0.789, 1.170)	3.569 (0.668, 6.555)	1.512 (0.621, 2.411)

1	0.375 (-0.030, 0.783)	-0.360 (-0.921, 0.204)	-0.078 (-0.588, 0.434)	0.343 (-1.128, 1.837)	1.126 (0.666, 1.589)

2	0.220 (-0.283, 0.725)	-0.469 (-1.168, 0.234)	-0.209 (-0.837, 0.423)	-1.250 (-3.076, 0.611)	0.664 (0.094, 1.237)

3	-0.011 (-0.396, 0.375)	-0.501 (-1.039, 0.039)	-0.207 (-0.689, 0.277)	-1.286 (-2.706, 0.155)	0.126 (-0.309, 0.564)

4	-0.316 (-0.965, 0.336)	-0.456 (-1.358, 0.455)	-0.072 (-0.889, 0.751)	0.233 (-2.140, 2.664)	-0.485 (-1.220, 0.255)

The remainder of this section describes more detailed results for the subset of the cases discharged with a primary diagnosis of heart failure. There were 170,502 emergency hospital admissions for heart failure after the exclusions, approximately 100 admissions per day, in the defined urban areas. Admission rates followed a seasonal cycle, approximately twenty percent higher during the winter compared to the summer. A long term trend was not evident. Geographically, the incidence of admissions based on the population over age 35 was highest in the inner city areas with high poverty rates. Table 2 summarizes characteristics of the population by study area. Estimates of the percent increase in hospitalizations per 10 ug/m^3 ^PM_2.5 _across days 0 to 2 ranged from a low of -3.9% (-9.2 to 1.7%) in Rochester to a high of 6.2% (0.4% to 12.3%) in Manhattan. The confidence intervals around these stratified estimates were large, and the estimates are considered to be homogeneous (I-statistic = 27%).

**Table 2:** Characteristics of the heart failure (HF) admissions by study area

	Manhattan	Staten Island	Bronx	Brooklyn	Queens	Nassau	Buffalo	Rochester
Number of HF admissions	6,820	5,598	40,767	34,251	24,141	32,724	16,534	9,667

Population age 35+	495,028	228,841	1,309,539	1,128,188	1,090,913	1,410,542	641,010	474,504

Age-adjusted rate/1000	13.7	26.2	30.7	29.9	21.7	23.4	22.5	19.7

HF as % of all cardiovascular admissions	24.0	22.6	30.0	27.9	24.5	24.0	25.5	26.8

Average age (years)	75.5	74.9	71.3	72.5	74.0	76.0	75.7	75.7

Male gender (%)	46.9	47.5	43.5	42.5	45.4	46.1	42.6	45.7

Additional diagnoses (%)								

ischemic heart disease	45.5	61.2	44.0	48.8	47.9	57.2	59.0	55.7

diabetes	36.8	43.1	45.4	41.4	41.6	38.6	40.8	41.1

COPD	27.3	32.5	31.4	28.5	25.8	30.3	37.8	31.9

conduction disorder	3.8	4.6	3.0	3.6	2.5	5.1	4.5	2.8

atherosclerosis	1.2	0.8	0.7	0.7	0.7	0.8	1.2	0.8

Previous HF admission (%)	38.6	38.8	43.5	35.6	31.4	34.8	31.8	31.6

Average % of adults living below poverty level	11.7	8.9	20.9	21.6	13.8	5.5	10.1	8.8

Average PM_2.5 _(ug/m^3^)	15.5	12.8	14.1	11.6	12.6	14.4	12.8	11.1

% increase HF admissions per 10 ug/m^3 ^PM_2.5 _lags 0 to 2 (95% conf. interval)	6.2 (0.4, 12.3)	5.9 (-1.1, 13.5)	3.3 (0.9, 5.7)	2.6 (0.0, 5.2)	4.7 (1.4, 8.1)	4.4 (1.4, 7.4)	4.8 (1.1, 8.6)	-3.9 (-9.1, 1.7)

An extreme air pollution episode occurred in July 2002, when hourly readings of PM_2.5 _reached over 130 ug/m^3 ^due to smoke from Canadian wildfires. Preliminary analysis showed that these extreme observations overly influenced the case-crossover model fit, with dfbetas (which measure changes in parameter estimates when the observation is omitted) more than ten standard deviations above the mean. The entire three day fire period was therefore set to missing to reduce the influence of these days and because the composition of the particulate matter during this period was different from the usual PM composition.

Estimates of daily PM_2.5 _using the combined TEOM and FRM dataset would have exceeded the new daily PM_2.5 _NAAQS of 35 ug/m^3 ^63 times out of 1,792 days (3.5%) in the study period in the New York City metropolitan area, 41 times (2.3%) in the Buffalo area and 16 times (0.9%) in the Rochester area. In case-crossover analysis, the power to detect an effect is related to the difference in exposure between the case and referents, not the absolute distribution of exposure observed during the study period [21]. We report the absolute difference between the exposure on the case day and the average of the exposure among the three referent days in Table 3. Average relevant differences for PM_2.5 _were approximately 6 ug/m^3^.

**Table 3:** Distribution of relevant differences in concentrations of PM_2.5 _and weather as measured in case-crossover analysis of heart failure, January 20, 2001 - December 16, 2005

	Percentile						
		
	Mean	5^th^	25^th^	50^th^	75^th^	95^th^	Max
PM_2.5 _(μg/m^3^)	5.8	0.4	2.1	4.5	8.0	16.0	42.2

Avg heat index (°C)	6.9	0.6	2.4	5.8	10.0	17.1	38.9

Avg air pressure (hPa)	6.6	0.4	2.5	5.4	9.3	17.5	34.9

μg/m^3 ^= micrograms per cubic meter

°C = degrees Celsius

hPa = hectaPascal

Table 4 summarizes the analysis of effect modification of PM_2.5 _on heart failure admissions by individual, area-level, and temporal characteristics. Older adults, particularly those age 85 and older, were significantly more susceptible to the short-term effects of PM_2.5 _than younger adults. The risk of hospitalization for individuals with atherosclerosis was almost three times greater than the risk for individuals without this condition, but this difference was not significant. The presence of other comorbid conditions was not related to increased risk. There was no significant relationship between poverty and susceptibility to PM_2.5_, although those living in areas with the least amount of poverty appeared slightly more susceptible to PM_2.5_. There was no statistically significant interaction between age, poverty, and PM_2.5_, though the power to detect such a difference is much smaller than the power to detect main effects.

**Table 4:** Modifications of the effect of ambient PM_2.5 _concentrations on heart failure admissions

	Description	n	% incr. risk^#^	LCLM^	UCLM^	p-value^!^
*Individual-level risk factors*						

Age	85+	39,538	6.47	3.98	9.02	0.03
	75-84	53,248	3.78	1.35	6.27	0.51
	65-74	36,690	2.52	-0.84	6.00	0.89
	35-64	41,026	2.76	0.36	5.22	(ref)

Gender	female	75,670	4.57	2.15	7.05	0.18
	male	94,832	3.02	1.15	4.93	(ref)

Atherosclerosis (ICD = 440)	present	1,389	10.66	-1.79	24.69	0.29
	absent	169,113	3.83	2.41	5.26	(ref)

Previous heart failure admission (ICD = 428)	present	62,043	4.51	2.03	7.05	0.41
	absent	108,459	3.52	1.88	5.20	(ref)

Ischemic heart disease (ICD = 410-414)	present	86,599	3.92	1.54	6.36	0.95
	absent	83,903	3.85	2.05	5.69	(ref)

Conduction disorder (ICD = 426)	present	6,238	3.66	-2.29	9.96	0.94
	absent	164,264	3.89	2.46	5.34	(ref)

Diabetes (ICD = 250)	present	70,986	3.72	1.31	6.18	0.80
	absent	99,516	4.01	2.31	5.72	(ref)

COPD (ICD = 490-496)	present	51,736	2.48	-0.05	5.07	0.10
	absent	118,766	4.51	2.91	6.14	(ref)

*Area-level risk factors*						

% adults living below poverty level, by census tract	20%-100%	57,593	2.45	-0.38	5.36	0.10
	7-20%	58,092	4.51	1.62	7.49	0.85
	0-7%	54,817	4.79	2.61	7.01	(ref)

*Temporal variables*						

Season	Cool	94,252	4.10	1.49	6.78	0.74
	Warm	76,250	3.66	1.75	5.61	(ref)

Year*	2005	34,141	2.9	-0.10	6.05	0.23
	2001	34,363	4.9	2.73	7.10	(ref)

# percent change in risk of heart failure admission per 10 ug/m^3 ^PM_2.5 _averaged over lags 0-2

*modeled as continuous variable

^ lower and upper 95% confidence limit around risk estimate

! p-value for significance of interaction term

As a sensitivity analysis, the base case-crossover model was repeated using a different referent selection method: 21-day strata with referents at +/- 7 and/or 14 days within each stratum as compared to original 28-day strata with referents at +/- 7, 14, and/or 21 days. Results (Table 5) are presented as both a relative risk and an attributable risk. The attributable risk is the proportion of emergency heart failure hospitalizations that would be eliminated if PM_2.5 _were reduced below a background of 5 ug/m^3^, assuming the exposure causes the outcome. This represents only the portion of the risk due to the acute effect of air pollution, the larger portion being due to chronic exposure [22]. Our results were sensitive to the selection of referents. On a relative scale, the risk obtained using the 21-day window was about half the risk estimated using the 28-day window. However, the confidence intervals around the estimates were large and overlapped.

**Table 5:** Sensitivity of results to referent selection method, shown as relative and attributable risk

	relative risk^# ^(95% CI)	attributable risk^! ^(95% CI)
28-day window	3.9 (2.5 to 5.3)	3.1 (2.0 to 4.1)

21-day window	2.1 (0.6 to 3.6)	1.7 (0.5 to 2.8)

^#^Percent change in risk of heart failure admission per 10 ug/m^3 ^PM_2.5 _averaged over lags 0-2.

^! ^Percent of admissions attributable to PM_2.5 _over background of 5 ug/m^3^

## Discussion

The health effects of air pollution observed in this analysis are consistent with the literature. The national study [3] that analyzed Medicare hospital admissions in 204 urban counties between 1999 and 2002 also found the strongest association with heart failure, with a 1.28% increase in risk per 10 ug/m^3 ^PM_2.5 _nationwide, and a risk closer to 2% in the Northeast. Since this estimate was based only on current day exposure, it underestimates the cumulative impact of PM_2.5 _that was distributed onto lags 1 and 2 as well. A review by the United Kingdom Committee on the Medical Effects of Air Pollutants showed that within studies comparing the association of various diagnoses to PM_10_, heart failure had the highest estimate in 4 out of 6 studies [23].

Several interrelated biologic mechanisms have been proposed to explain how inhalation of particulate matter may result in cardiopulmonary morbidity and mortality [2]. The increased risk among those with heart failure supports the role of altered cardiac autonomic function, and the increased risk among those with peripheral artery disease supports the role of inflammation-accelerated atherosclerosis [23].

Susceptibility to PM_2.5 _increased with increasing age, and was strongest in those over 85. Our analysis also included cases under age 65, which diluted the overall effect estimate and reduced the chances of observing effect modification by factors other than age. It did, however, show a small increased risk among heart failure cases under age 65.

Area level poverty was not a significant effect modifier, however, we observed that cases living in areas with a higher percentage of people living under the poverty level were slightly less susceptible to air pollution. A review of the effects of socioeconomic status on the relationship between air pollution and mortality [10] found that effect modification was more likely to be identified as geographic resolution increased: city or county-level studies found no modification, those using finer areas found mixed results, and those using individual level data most consistently found that pollution more strongly affected those of lower socioeconomic status. There could be several reasons for the opposite relationship we observed. First, area level poverty rates may not accurately reflect the individual risk. There may have been a bias in exposure estimation since housing in high poverty areas may be situated closer to traffic, yet neighborhoods with high and low poverty levels were assigned the same air pollution values. Competing risks could also have played a role. Neighborhoods with higher poverty rates had younger cases on average, and younger people are generally less susceptible to the cardiovascular effects of air pollution. The older, wealthier cases may appear to be more susceptible to air pollution if they have lived longer and don't have other risk factors for heart failure. Medical coding differences may have also influenced the results. Blacks with concurrent heart failure, hypertension, and myocardial infarction were shown to be more likely to receive a primary diagnosis of heart failure than myocardial infarction [24]. Lastly, we only modeled the impact of poverty on PM_2.5_, ignoring subtle effect modification by the other confounding variables in the model.

For heart failure cases with atherosclerosis, the risk of hospitalization increased 10.7% per 10 ug/m^3 ^PM_2.5_, compared to 3.8% for those without this comorbid condition. This estimate was the large in terms of magnitude, but imprecise due to the relatively small number of people with this condition (n = 1,389).

The point estimates in this analysis were sensitive to the referent selection methodology, though the confidence intervals around the estimates were large compared to the magnitude of the effect. Similar sensitivity results were observed within the Poisson time-series method in an analysis of the robustness of the models to control for weather [25]. The investigators found that the lowest effect estimate of the models was 58% of the magnitude of the highest effect estimate, again within the range of the confidence intervals.

There are several limitations to this analysis. As with all population-based air surveillance analyses, we assume that air pollutant concentrations measured at a community level serve as a surrogate for the average personal exposure during the respective time period. Factors such as time spent in traffic, indoor air emission sources, and smoking affect the validity of this assumption [26]. Temporal misclassification of exposure likely resulted in bias towards the null. In this study, exposures were assigned based on 12:00 am to 11:59 pm daily averages, such that for individuals admitted at 1 am, the assigned 24 hour average "current day" exposure would have actually occurred after admission. Symons et al. [12] found that associations based on an 8-hour lagged exposure period were stronger than associations based on 24-hour lagged exposure. Since hour of admission is available on the NYS hospital discharge dataset, future analyses could link the health and air quality on the hourly scale rather than the daily scale. Challenges in hourly analysis, though, include the need to correct for the larger loss of semi-volatiles in the winter by hour (rather than simply by day), and the need for a more flexible method such a splines to characterize the shape of the distributed lag function [13]. Hourly or true-lagged exposure analyses would be most useful for a disease such as atherosclerosis that was shown in the daily-level analysis to have an acute effect on the current day. Future analyses would also benefit from adjustment for gaseous criteria pollutants which might slightly affect PM risk estimates [1]. Comparisons of the effects of air pollution across disease groups may have been affected by misclassification, as coding may be affected by administrative hospital decisions [24,27]. A more substantive problem is that surveillance methods, including both case-crossover and Poisson time series, can only estimate the short-term effect of air pollution, thus underestimating the combined acute and chronic effects that impact population health [22].

## Conclusion

PM_2.5 _had a stronger impact on heart failure than other cardiovascular diagnoses, with 3.1% of heart failure admissions attributable to short-term PM_2.5 _over background levels of 5 ug/m^3^. Older adults were most susceptible to heart failure after short-term ambient PM_2.5 _exposure. There were no significant differences by geographic area, poverty level, or comorbid conditions. Daily PM_2.5 _estimates from the TEOM data were important for estimating a distributed lag model, as the effect of PM_2.5_lasted for several days.

Environmental public health practitioners and policy analysts need high quality, consistent data and tools to assess the effectiveness of interventions intended to reduce the impacts of air pollution. However, given the relatively small risk associated with air pollution, the uncertainty around that risk, the geographic heterogeneity of populations, and the many other time varying factors that influence morbidity rates, tracking changes in time and/or space is difficult. The Environmental Public Health Tracking Program is currently assembling nationally consistent air pollution and health outcome data to facilitate tracking the health impacts of air pollution.

## List of Abbreviations

DOH: Department of Health; EPHT: Environmental Public Health Tracking; NAAQS: National Ambient Air Quality Standards; NYS: New York State; NYC: New York City; SES: socioeconomic status; FRM: Federal Reference Method; NCDC: National Climatic Data Center; TEOM: Tapered Element Oscillating Microbalance; ZCTA: ZIP code tabulation area.

## Competing interests

The authors declare that they have no competing interests.

## Authors' contributions

VH participated in the design of the study, prepared the datasets, performed the statistical analysis, and drafted the manuscript. TT participated in the design and coordination, and helped to draft the manuscript. HF helped with the analysis and interpretation of the air pollution data. All authors read and approved the manuscript.
